# Identification of Monotonically Differentially Expressed Genes across Pathologic Stages for Cancers

**DOI:** 10.1155/2020/8458190

**Published:** 2020-11-12

**Authors:** Suyan Tian, Chi Wang, Mingbo Tang, Jialin Li, Wei Liu

**Affiliations:** ^1^Division of Clinical Research, The First Hospital of Jilin University, 1 Xinmin Street, Changchun 130021, Jilin, China; ^2^Department of Internal Medicine, College of Medicine, University of Kentucky, 800 Rose Street, Lexington 40536, KY, USA; ^3^Markey Cancer Center, University of Kentucky, 800 Rose Street, Lexington 40536, KY, USA; ^4^Department of Thoracic Surgery, The First Hospital of Jilin University, 1 Xinmin Street, Changchun 130021, Jilin, China

## Abstract

Given the fact that cancer is a multistage progression process resulting from genetic sequence mutations, the genes whose expression values increase or decrease monotonically across pathologic stages are potentially involved in tumor progression. This may provide insightful clues about how human cancers advance, thereby facilitating more personalized treatments. By replacing the expression values of genes with their GeneRanks, we propose a procedure capable of identifying monotonically differentially expressed genes (MEGs) as the disease advances. Using three real-world gene expression data that cover three distinct cancer types—colon, esophageal, and lung cancers—the proposed procedure has demonstrated excellent performance in detecting the potential MEGs. To conclude, the proposed procedure can detect MEGs across pathologic stages of cancers very efficiently and is thus highly recommended.

## 1. Introduction

Since cancer is a multistage progression process that results from genetic sequence mutations, the patterns of gene expression values differ as tumors develop. “Monotonic” genes, whose expression levels increase or decrease monotonically as the disease advances, are highly likely to be involved in the tumor progression. Therefore, they may provide insightful clues about how these complex diseases initiate and advance and have potential to facilitate personalized treatments. Thus, the roles they play in cancers are of critical importance.

Feature selection is one of the fundamental tasks in the area of machine learning. Generally speaking, the primary objective of feature selection is to identify an optimal subset of genes associated with the phenotype(s) of interest. Therefore, identification of genes presenting some specific expression change patterns over pathologic stages is essentially a process of feature selection. So far, only a few feature selection algorithms that are capable of identifying monotonically differentially expressed genes (MEGs) across time points/stages have been proposed.

The MFSelector method proposed by Wang et al. [1] and the pipeline to analyze longitudinal big data proposed by Carey et al. [2] are two such methods. Briefly, the MFSelector method selects *K*-1 (where K is the number of stages/time points under consideration) discriminating lines to separate the stages or time points apart and calculates a statistic (the number of misclassified subjects according to the *K*-1 discriminating lines), and the *p* value determines whether the specific gene is monotonically differentially expressed. In the analysis pipeline by Carey et al. [2], the functional principal component analysis is used to fit a smooth curve for longitudinal expression values of each gene. Then, modified *F*-tests are carried out to screen the genes according to the corresponding *p* values of *F*-statistics. Clustering is conducted to group the statistically significant genes according to whether they are co-expressed. These resulting clusters are called as gene response modules in which the monotonically increasing and the monotonically decreasing patterns over time are the primary patterns of concern.

The MFSelector method and the longitudinal data analysis pipeline are conventional feature selection methods and do not take pathway information into account. Many studies have demonstrated that more advanced feature selection methods in which pathway information is incorporated as a priori to guide the process of feature selection outperform those classic feature selection methods in terms of predictive capacity, model stability, and biological implication. Such advanced feature selection algorithms are referred to as pathway-based feature selection algorithms [3]. As we mentioned in our previous studies [3, 4], the weighting strategy is the simplest way to account for pathway information and, as long as the estimation of those weights is accurate enough, the strategy can have an excellent performance and in many cases outperforms the competitive methods.

In this study, we replaced the original gene expression values with the weighted expression values generated by the GeneRank method [5] and suggested a procedure to identify MEGs. The method was evaluated using three sets of real-world gene expression data and the results were compared with the conventional method using original gene expression values and the MFSelector method [1].

## 2. Materials and Methods

### 2.1. Experimental Data

#### 2.1.1. Non-Small-Cell Lung Cancer (NSCLC)

The raw data of the NSCLC studies we used are stored on the Gene Expression Omibus (GEO: https://www.ncbi.nlm.nih.gov/geo/) repository under accession numbers GSE37745 [6] and GSE50081 [7] and are publicly assessable. The chips of these two experiments were all profiled on the Affymetrix HG-U133 Plus 2.0 platform. All patients in these two cohorts were adjuvant treatment naïve with their survival time available. In our previous study [8], we gave a detailed description on this data set. Briefly, of 104 patients in the dataset, 17 were stage IA patients, 57 stage IB patients, 5 stage IIA patients, and 25 stage IIB patients.

#### 2.1.2. Colon Cancer (CC)

The accession number on the GEO repository for the colon cancer data considered in this study is GSE62932 [9]. The chips of this experiment were hybridized on the Affymetrix HG-U133 Plus 2.0 platform as well. The data include 4 normal controls, 12 stage I patients, 17 stage II patients, 20 stage III patients, and 15 stage IV patients, for a total of 68 subjects in this study.

#### 2.1.3. Esophageal Cancer (EC)

The RNA-Seq data of the Cancer Genome Atlas Data Portal Esophageal Carcinoma (ESCA) cohort were downloaded from Genomic Data Commons (https://gdc.cancer.gov/). Patients with no clinical information on their pathologic stage were excluded, leaving 145 patients to be considered in the downstream analysis. Among them were 17 stage I patients, 70 stage II patients, 50 stage III patients, and 8 stage IV patients.

### 2.2. Preprocessing Procedures

Raw data (.cel files) of the three microarray datasets were downloaded from the GEO repository. The expression values were obtained using the fRMA algorithm [10] and were normalized using quantile normalization. For the NSCLC data, after the summary expression values were obtained, the COMBAT algorithm [11] was used to eliminate or alleviate the potential batch effects existing among different experiments.

For the RNA-Seq data of esophageal cancer, FPKM was downloaded from Genomic Data Commons (https://gdc.ca-ncer.gov/). The gene expression values were obtained by adding ones to FPKM counts and then having them log 2 transformed.

### 2.3. Pathway Information

The interaction/connection information among genes was retrieved from the Human Protein Reference Database (HPRD) [12], and the adjacency matrix was made on the basis of these gene-to-gene interactions. There were 9,672 protein-coding genes annotated in the HPRD database, Release 9 (http://www.hprd.org/).

### 2.4. Statistical Methods

#### 2.4.1. GeneRank

Briefly, the GeneRank *r* for gene *i* is solved by

(1) $$\begin{array}{c} \left(I_{p}-dWD^{-1}\right)r=\left(1-d\right)\exp_{i}. \end{array}$$

In this equation, *I*_*p*_ is a *p*×*p* identity matrix. Here, *p* is the number of genes under consideration; *W* stands for the adjacency matrix of genes and records how they interplay with one another, if the value in its *kj* cell is 1 then gene *k* and gene *j* are connected, and the value is zero otherwise. *D* is a *p*×*p* matrix, with its diagonal elements recording the degrees of freedom for these *p* genes and off-diagonal elements are zeroes. The degree of freedom is the number of genes to which a specific gene k (*k* = 1,2,…, *p*) is connected; expi stands for gene expression values for sample i (*i* = 1,2,…, *n*), and *d* is a damping or tuning parameter, balancing off the influence of the expression values and the pathway topological information within the network on the final rankings. The rankings can be completely determined either by the expression values when *d* equals to 0 or by the network structure when *d* equals to 1. The value of *d* is set at 0.5 by default.

### 2.5. Monotonic Expression Pattern Identification

The procedure we propose consists of three steps. First, the GeneRank of each gene for each subject is generated. Second, upon those GeneRanks that may be regarded as the weighted expression values of genes, the Kruskal-Wallis tests are carried out. Genes with adjusted *p* values less than a predetermined threshold (here, a grid of values are considered, i.e., 0.05, 0.1, 0.15, and 0.2) are deemed to be statistically differentially expressed genes. Among those differentially expressed genes, different expressed patterns such as a U-shaped relationship or a spike at a single stage are possible but not of interest. Thus, the following equations are further exploited to distinguish monotonic expression patterns from other patterns:

(2) $$\begin{array}{c} \left({\bar{\exp}}_{i0}\leq \right){\bar{\exp}}_{i1}\leq {\bar{\exp}}_{i2}\leq {\bar{\exp}}_{i3}\leq {\bar{\exp}}_{i4}, \end{array}$$

(3) $$\begin{array}{c} \left({\bar{\exp}}_{i0}\geq \right){\bar{\exp}}_{i1}\geq {\bar{\exp}}_{i2}\geq {\bar{\exp}}_{i3}\geq {\bar{\exp}}_{i4}, \end{array}$$

for the monotonically increasing (MI) expressed genes and the monotonically decreasing (MD) expressed genes, respectively. Here, ${\bar{\exp}}_{i0}$stands for the mean expression value of gene *i* in the normal control group. Notably, it is put inside parentheses to emphasize that not all studies have included controls. Furthermore, ${\bar{\exp}}_{ik}$stands for the mean expression value of gene *i* among the patients at pathologic stage *k* (*k* = 1, 2, 3, and 4). Specifically, for the colon cancer and esophageal cancer studies, it corresponds to stages I II, III, or IV, and for the NSCLC study, it corresponds to stages IA, IB, IIA, or IIB.

### 2.6. Kruskal-Wallis Test

Kruskal-Wallis tests are carried out to determine if any differences in expression values exist among different stages, and then the differentially expressed genes presenting monotonic expression patterns are selected by equations (2) and (3). So, the only difference between this procedure and the procedure we propose is that the conventional one uses original expression values, whereas the proposed one uses weighted expression values generated by the GeneRank method.

### 2.7. MFSelector

Another method capable of identifying MEGs is the MFSelector method [1] in which a new statistic, the DEtotal (total discriminating error) score is introduced, and the corresponding adjusted *p* value which corrected for the multiple comparisons problem of the DEtotal score is calculated using permutation tests. Using the monotonically increasing scenario to illustrate the MFSelector method is described briefly as follows.

For a monotonically increasing expressed gene, it is naturally expected that subjects in early stages have smaller expression values compared to the subjects in later stages. First, *n*1 (*n*1 is the number of patients at stage I) discriminating lines may be drawn at the expression value of each stage I patient. The stage I patients above this line and the patients at higher levels below this line are misclassified, and the number of misclassified patients is counted. The final discriminating line to separate stage I from the higher levels corresponds to the line with the least misclassified number. This step is repeated for *K*−1 times to discriminate the patients at the first *k* (*k* = 1,2,…, *K*−1, where *K* is the total number of stages) stages from the remaining patients, resulting in *K*−1 discriminating lines. If a gene has *K*−1 distinct discriminating lines and the lines for a later stage are above the lines for an earlier level, the expression change pattern of this gene has a perfect monotonically increasing expression tendency. Then, the DEtotal score is the sum of misclassified numbers for the *K*−1 segmentations, and a *p* value/*q* value of the DEtotal score is calculated using permutation tests (the patient's labels are perturbed) to determine whether or not this specific gene's increasing expression is statistically significant.

## 3. Results

### 3.1. Identification of MEGs

Colorectal cancer (CC), also known as colon cancer, is the second most common cancer in females and the third in males [13]. The molecular mechanisms of colon cancer have not yet been fully elucidated [14]. Likewise, the underlying mechanisms for esophageal cancer have not been unraveled, but the incidence and mortality rates are lower compared to colon cancer. For both sexes combined, lung cancer is the most commonly diagnosed cancer and the leading cause of cancer death [13]. Even though much more research is done on lung cancer compared to colon and esophageal cancers, complete deciphering of its etiology and progression has not yet been achieved.

Since the colon and esophagus both belong to the gastrointestinal tract, they may share more similarities regarding gene expression compared to lung cancer; thus, the MEGs for colon and esophageal cancers are expected to have more overlap with each other than with lung cancer. On the other hand, the platforms of the colon cancer and esophageal cancer studies differ, while the platforms for the NSCLC study and colon cancer study are identical, even though the origin of NSCLC is the respiratory system rather than the gastrointestinal tract. Moreover, esophagus and lung are located inside the thoracic cavity and the colon is inside the abdominal cavity. With these similarities and dissimilarities, the three data sets may disclose many interesting patterns. Utilizing the GeneRank method [5] as a building block, we propose a procedure that enables identification of monotonically expressed genes (MEGs) in this study, with the objective of revealing underlying molecular mechanisms for these three cancers.

The numbers of selected monotonically expressed genes over stages for these three types of cancers using the proposed procedure are given in Table 1, with the significant levels set at 0.05, 0.1, 0.15, and 0.2, respectively. In addition, the number of MEGs by the conventional Kruskal-Wallis method using the unweighted expression values and the MFSelector method (described briefly in the Methods section) are given in Table 1. Compared to the proposed method, both the Kruskal-Wallis method and the MFSelector method are too conservative, especially when using the MFSelector method, as no genes were identified as MEGs for any of the three studies for any of the significant levels considered. Therefore, the proposed procedure is decidedly more statistically powerful in detecting potential MEGs.

Interestingly, we observed the following tendency—for both esophageal cancer and NSCLC, the number of monotonically increasing genes is larger than that of monotonically decreasing genes. On the other hand, the opposite case is true for colon cancer, which may imply that more potential tumor suppressor genes are off balance for colon cancer, whereas more potential oncogenes are off to boost tumor progression for both esophageal cancer and NSCLC. Further investigation is warranted.

**Figure 1:**
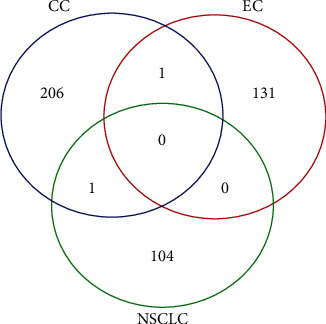
Venn diagram of the identified MEGs for colon cancer, esophageal cancer, and non-small-cell lung cancer studies. CC: colon cancer; EC: esophageal cancer; NSCLC: non-small-cell lung cancer. MEGs: monotonically expressed genes.

With the cutoff for adjusted *p* value set at 0.1, a Venn diagram of identified MEGs for the three studies using the proposed procedure is shown in Figure 1. It is observed that one overlap, i.e., COMMD7 (COMM domain containing 7) existed in the MEGs for colon and esophageal cancer, whereas the other overlap, i.e., HAND2 (heart and neural crest derivatives Expressing 2) existed in the MEGs for colon cancer and NSCLC.

**Figure 2:**
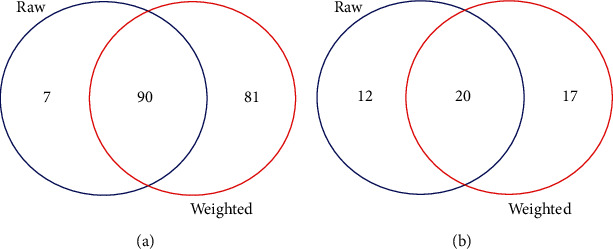
Comparison of the identified MEGs for colon cancer by the conventional method and the proposed procedure. (a) For the monotonically decreasing genes. (b). For the monotonically increasing genes. The significance level is set at 0.1. MI: monotonically increasing; MD: monotonically decreasing; raw: the MEGs identified by the conventional method upon the original expression profiles; weighted: the MEGs identified by the proposed method upon the weighted expression profiles; MEGs: monotonically expressed genes.

**Figure 3:**
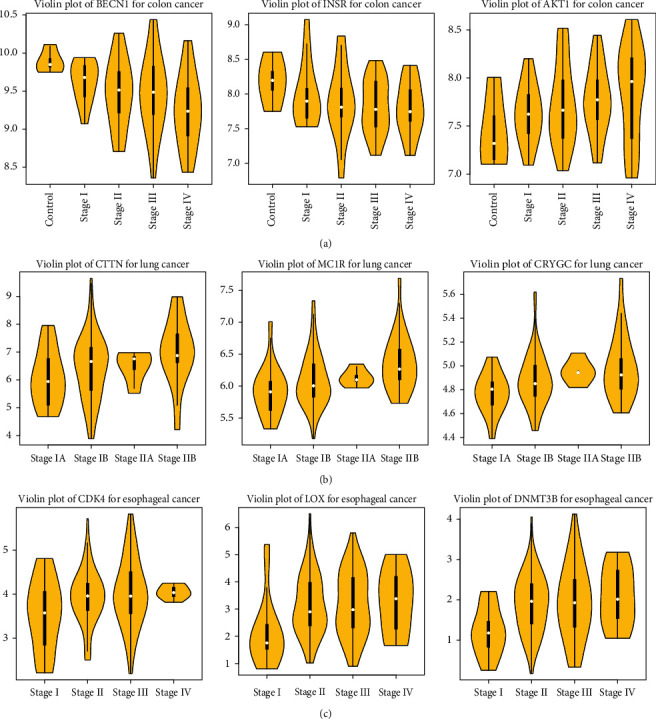
Violin plots of three randomly selected MEGs for each cancer type. (a) Colon cancer. (b) Esophageal cancer. (c) Non-small-cell lung cancer. CC: colon cancer; EC: esophageal cancer; NSCLC: non-small-cell lung cancer. MEGs: monotonically expressed genes.

A comparison between the proposed procedure and the conventional method for the colon cancer study was also made. The results are presented in Figure 2. The Venn diagrams stratified by the expression direction show that the overlap of MEGs by these two methods is substantial. The resulting weighted expression values balance between gene expression values and their importance (i.e., the degree of connectivity) in the gene-to-gene interaction network, thus MEGs identified by the proposed procedure alone tend to be essential genes in the network. Using the conventional method, these genes would be left out due to their subtle expression levels. For each cancer type, three MEGs were randomly selected, and violin plots representing their expression distributions stratified by pathologic stage are shown in Figure 3. Basically, no too extreme values are detected in the expression levels of the nine genes.

**Figure 4:**
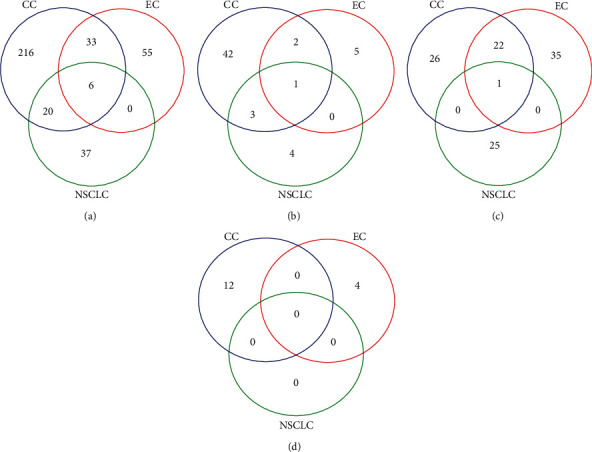
Venn diagrams of the enriched GO terms and KEGG pathways by the MEGs for colon cancer, esophageal cancer, and non-small-cell lung cancer studies. (a) GO biological process terms. (b) GO molecular function terms. (c) GO cellular component terms. (d) KEGG pathways. CC: colon cancer; EC: esophageal cancer; NSCLC: non-small-cell lung cancer, BP: biological process; MF: molecular function; CC: cellular component.

The enriched gene ontology (GO) terms [15] and KEGG pathways [16] by the MEGs were explored using the String software, stratified by each study. For NSCLC, there are 63 enriched GO biological process terms, 8 GO molecular function terms, 26 GO cellular component terms, and 0 KEGG pathways, respectively. For esophageal and colon cancers, the numbers of enriched GO terms by identified MEGs are 94 and 275 biological process terms, 8 and 48 molecular function terms, 58 and 49 cellular component terms, and 4 and 12 KEGG pathways, respectively. The Venn diagrams of overlapping GO terms and KEGG pathways are shown in Figure 4. Overall, at the gene set/pathway level, the overlap rate is higher than it is at the individual gene level, as expected.

### 3.2. Biological Relevance

#### 3.2.1. Overlapping MEGs

A recent study [17] claimed that COMMD7 overexpression positively correlated with histological differentiation and tumor node metastasis (TNM) stage of pancreatic ductal adenocarcinoma (PDAC), and PDAC patents with higher COMMD7 expression tended to have poorer overall survival rates. Also, COMMD7 has been reported to be upregulated in hepatocellular carcinoma (HCC) and promote HCC cell proliferation [18]. Even though in the literature we cannot find any studies suggesting COMMD7 is explicitly associated with esophageal or colon cancer, the proposed method identified it as a monotonic increasing gene for both EC and CC cohorts, consistent with the results of the two abovementioned studies and supporting the thought that COMMD7 is an oncogene. In contrast, another recent study [19] showed that HAND2 was hypermethylated and downregulated in colon cancer, while another study [20] demonstrated that HAND2 was overexpressed in the lung squamous cell carcinoma. In the present study, HAND2 was identified as a monotonically decreasing gene in colon cancer while a monotonically increasing gene in NSCLC, which is basically consistent with the results of the two previous studies.

#### 3.2.2. Type-Specific MEGs

MEGs that are specific for one cancer type, meaning the genes were identified as the MEGs by only a single study, are referred to as type-specific MEGs. According to the GeneCards database, all these genes are related to cancer either directly or indirectly. Some of them have been demonstrated to associate with these three cancer types under investigation by experimental means. For example, AKT1 (AKT serine/threonine kinase 1) has been demonstrated to play crucial roles in the development, progression, and drug resistance of colon cancer [21, 22]. In addition, Zhao et al. [23] showed that MiR-124 was significantly downregulated in NSCLC patients, and miR-124 negatively regulates AKT1. As far as esophageal cancer is concerned, the expression level of AKT1 has been reported to be significantly elevated in tumor tissue of patients with esophageal squamous cell carcinoma [24].

For esophageal cancer, the GeneCards database ranks CDK4 (cyclin-dependent kinase 4), DNMT3B (DNA methyltransferase 3 beta), and MAGEA4 (MAGE family member A4) as the top three relevant genes. Among the 24 MEGs that are directly related to esophageal cancer, a majority of them are associated with either NSCLC or colon cancer. For example, LOX (lysyl oxidase) has been shown to be overexpressed in lung cancer, and inhibition of LOX activity decreases the number of lung metastases [25].

For NSCLC, the GeneCards database indicates that 23 MEGs are directly related to lung cancer. ESR2 (estrogen receptor 2), CHKA (choline kinase alpha), and CRYGC (crystallin gamma C) are identified as the top three NSCLC-specific MEGs. ESR2 and CHKA are also associated with colon and esophageal cancers, while CRYGC is only directly related to colon cancer according to the GeneCards database. Even though type-specific genes were only identified as MEGs by a single study, many of them were correlated with the other two cancer types.

#### 3.2.3. Oncogenes or Tumor Suppressor Genes?

For the top MEGs with good biological relevance (i.e., the genes have a confidence score of >5 in the GeneCards database), whether the certain genes are oncogenes or tumor suppressor genes was investigated by searching the PubMed database and the TSGene 2.0 [26] database which records tumor suppressor genes for about 10 cancer types including colon cancer, lung adenocarcinoma, and lung squamous cell carcinoma.

For the top colon cancer MEGs, the consistent tumor suppressor genes included MAP2K4, MAPK10, RUNX3, WNK2 (the four genes were identified by the TSGene 2.0 database), BECN1 [27], FASN [28], NAT1 [29], and NR3C2 [30]. For monotonically increasing genes, the consistent ones include CCKBR, BMP4 [31, 32], and SLC29A1 [33] which were determined to be oncogenes by previous studies. In contrast, RB1 that was regarded as a tumor suppressor gene [34] in gastrointestinal stromal tumors and was identified as a monotonically increasing gene, while three oncogenes including NFE2L2 [35], ABL1 [36], and LASP1 [37] were identified as monotonically decreasing genes. Of note, AKT1 is indicated as a tumor suppressor gene by the TSGene 2.0 database [26], but many previous studies (e.g., [38]) report it as an oncogene, as does the present study.

Three lung cancer MEGs had a confidence score of >5: ESR2 and CRYGC (monotonically increasing) and CHKA (monotonically decreasing). A meta analysis [39] found no association between ESR2 expression level and the prognosis of NSCLC patients, and thus whether it is an oncogene or not remains controversial. For CHKA, previous studies present contradicting results, for example, [40] indicated its expression was lower, while [41] mentioned it was overexpressed in lung cancer. No literature about the expression status of CRYGC in lung cancer was found. In addition, there are some inconsistencies between our work, the literature, and the TSGene 2.0 database. Specifically, CDH4 [42], SFRP1 [43], and ERF [44] which are indicated to be tumor suppressor genes by the TSGene 2.0 database and have support from the literature as well; however, our method identified them as monotonically increasing genes. These genes may be false positives by our approach. Lastly, the roles of several genes play remain controversial. Namely, NFATC2 is indicated to be a tumor suppressor by the TSGene 2.0 database. However, Xiao's study [45] suggested high expression associated with poor tumor differentiation and poor survival. Similarly, a recent study [46] showed that the expression of AHNAK was upregulated in tumor samples, while the TSGene 2.0 database deems it as a tumor suppressor gene. The present study identified these two genes as monotonically increasing genes, being consistent with the previous studies.

Lastly, for the three monotonically increasing genes with good biological relevance, CDK4, DNMT3B, and MAGEA4 to esophageal cancer, previous studies [47, 48] suggested the last two genes as oncogenes for esophageal cancer while another study [49] suggested CDK4 was underexpressed in the tumor samples of esophageal cancer. The heterogeneity of study population, experimental techniques and personnel, and so on may explain the inconsistencies and contradictions to some extent. Further investigation on the roles that identified MEGs may play is highly desirable, especially for ones that are newly discovered by the proposed procedure.

## 4. Conclusions

After replacing the original expression values of genes with their GeneRanks [5], we defined a procedure capable of identifying genes with monotonically changed expression patterns across the pathologic stages of cancers. Using three real-world datasets, we show that the proposed method is superior to the conventional Kruskal-Wallis test and the MFSelector method [1]. Furthermore, the MEGs we identified are highly associated with the development and prognosis of cancer.

This procedure should be applicable to not only mRNA data but also many other data types such as lncRNA (long noncoding RNA) data. For the noncoding RNAs, there is no canonical knowledge base, such as STRING [50] and HPRD [12], to record how they interact. Furthermore, given the mechanism of how LncRNAs impact on a biological process by acting as a miRNA sponge, via the strategy of competing endogenous RNAs (ceRNAs) [51], the lncRNA-miRNA-mRNA interaction network may be more desirable. To address this shortage, statistical methods such as the WGCNA method [52] may be utilized to construct a data-driven gene-to-gene interaction network, upon which the importance of specific lncRNAs and their expression patterns over pathologic stages can be inferred.

To conclude, the gain of efficiency in detecting MEGs using the proposed procedure is nontrivial; therefore, it is highly recommended.

## Figures and Tables

**Table 1:** Monotonically differentially expressed genes.

Study		0.05	0.1	0.15	0.2
Colon cancer (*n* = 68)	MI	1 (0/0)	37 (32/0)	78 (81/0)	114 (127/0)

4 stages (I, II, III, and IV) and controls	MD	31 (0/0)	171 (97/0)	245 (157/0)	278 (204/0)

Esophageal cancer (*n* = 145)	MI	0 (0/0)	119 (0/0)	304 (0/0)	456 (25/0)

4 stages (I, II, III, and IV)	MD	0 (0/0)	13 (0/0)	32 (0/0)	54 (3/0)

NSCLC (*n* = 104)	MI	0 (0/0)	102 (0/0)	266 (0/0)	342 (0/0)

4 stages (IA, IB, IIA, and IIB)	MD	0 (0/0)	3 (0/0)	7 (0/0)	20 (0/0)

MI: monotonically increasing expressed genes; MD: monotonically decreasing expressed genes; NSCLC: non-small-cell lung cancer. (x/xx): x is the number of MEGs identified by the conventional Kruskal-Wallis method and xx is the number of MEGs identified by the MFSelector method. For example, for the NSCLC application at the significance level of 0.1, the (0/0) entity after 102 means both the conventional method and the MFSelection method identified 0 MI genes.

## Data Availability

Three microarray data (accession numbers: GSE37745, GSE50081, and GSE62932) were downloaded from the Gene Expression omnibus (GEO) repository (https://www.ncbi.nlm.nih.gov/geo/), and the RNA-Seq data for the ESCA cohort were downloaded from the Cancer Genome Atlas data portal (https://tcga-data.nci.nih.gov/tcga/). They are all free to be downloaded.
